# Study on Comprehensive Quality Control of Herba Hyssopi Based on Chemical Components and Pharmacological Mechanism Action

**DOI:** 10.3390/molecules31020205

**Published:** 2026-01-06

**Authors:** Zhenxia Zhao, Jiangning Peng, Yingfeng Du, Xinyi Yang, Lilan Fan, Cong Li, Amatjan Ayupbek, Hui Li, Yongli Liu

**Affiliations:** 1 Hebei Institute of Drug and Medical Device Inspection, Shijiazhuang 050027, China; zhaozhenxia@hbyxjy.org.cn (Z.Z.); 23034101844@stu.hebmu.edu.cn (J.P.); yaxy1408@163.com (X.Y.); yjs20244011@hebcm.edu.cn (L.F.); licong@hbyxjy.org.cn (C.L.); 2 College of Pharmacy, Hebei Medical University, Shijiazhuang 050017, China; 17700941@hebmu.edu.cn; 3 School of Pharmacy, Henan University, Kaifeng 475004, China; 4 School of Pharmacy, Hebei University of Chinese Medicine, Shijiazhuang 050200, China; 5 NMPA Key Laboratory for Quality Control of Traditional Chinese Medicine, Uyghur Medicine, XinJiang Uygur Autonomous Region Institute for Drug Control, Urumqi 830054, China; hefengzhulu2025@126.com

**Keywords:** UPLC-LTQ-Orbitrap-MS, network pharmacology, cellular experiments, cough and asthma, multivariate chemometric analysis

## Abstract

Herba Hyssopi is a key remedy in Uighur medicine for asthma and cough, frequently used as the monarch or minister herb in prescriptions. However, the lack of effective quality assessment methods complicates the detection of adulteration with common substitutes. In this study, UPLC-LTQ-Orbitrap-MS, network pharmacology, molecular docking, and cell experiments were employed to establish scientific and effective quality control methods to differentiate *Hyssopus cuspidatus Boiss* from its common adulterants. The results showed that a total of 41 chemical constituents were identified from Herba Hyssopi. Network pharmacology analysis revealed 133 potential target genes associated with its therapeutic actions, among which EGFR, MMP9, TNF, PTGS2, MAPK3, ESR1, and TP53 emerged as key targets. Cellular experiments further demonstrated that diosmin, linarin, and rosmarinic acid significantly suppressed nitric oxide (NO) generation and the release of pro-inflammatory cytokines. Based on these findings, a validated HPLC method was established for the simultaneous quantification of these three bioactive markers, providing a reliable tool for the quality assessment and authentication of Herba Hyssopi. This study offers a scientific basis for improving the standardization and quality control of Herba Hyssopi in traditional medicine applications.

## 1. Introduction

Respiratory diseases such as cough and asthma, influenced by environmental factors, among others, are a significant public health concern in China [1,2]. These diseases often require long-term medication, but 5–10% of patients may not respond well to such treatments [3], which can also cause adverse effects with prolonged use [4,5]. Botanical medicines such as Herba Hyssopi are increasingly used for chronic conditions due to their natural origin, mild effects, and low toxicity [6,7]. This article presents a scientific and efficient quality control approach for Herba Hyssopi, based on an investigation of the material foundation and pharmacological mechanism of the herb.

Herba Hyssopi, or “Zufa” in Uyghur medicine, is the dried aerial part of the *Hyssopus cuspidatus Boiss* (HCB) from the Lamiaceae family. Traditionally utilized in ethnic folk medicine [8], it is employed for medicinal purposes, primarily distributed in Xinjiang (China), Mongolia, Kazakhstan, and Russia [9]. It contains flavonoids, volatile oils, and organic acids, etc. [10,11]. The herb is used to treat respiratory issues, such as colds, coughs, asthma, and fever, by warming the lungs, dispelling cold, and reducing inflammation. It is especially significant in Uyghur medicine for addressing cold-type respiratory disorders with congestion and phlegm [12,13].

Herba Hyssopi is documented in several authoritative compilations, including Xinjiang Chinese Medicinal Materials, Uyghur Materia Medica, Flora Xinjiangensis (Volume 4), and the Chinese Herbal Medicine (Uyghur Medicine Volume), with the botanical origin consistently identified as HCB. It is recognized as one of the “Fifty Signature Medicinals” of Xinjiang and serves as a primary ingredient in Uyghur formulations such as Hanchuan Zupa Granules and Loki Zupa Compound [8,14].

Despite its significant medicinal value, the herb faces challenges, including botanical misidentification, supply shortages, and outdated quality standards, which compromise comprehensive quality control. These factors have led to market irregularities where cultivated substitutes (*Hyssopus officinalis* L.) and adulterants (*Nepeta bracteata* Benth) are frequently introduced into medicinal use [15,16]. As a result, the development of a scientifically rigorous quality control methodology is essential.

Although some preliminary studies have explored quality control methods for HCB, including content determination for components such as Rutin, Oleanolic acid, Chlorogenic acid, Rosmarinic acid, Quercetin, Luteolin, and Lcacetin [17,18,19,20], the correlation between these components and the efficacy of Herba Hyssopi has not been investigated. Furthermore, it remains unclear whether the current quality control methods can differentiate between authentic and adulterated products. The majority of natural medicines have a limited connection to safety and efficacy, which limits their clinical utility and hinders the modernization of traditional Chinese medicine. However, identifying key components is essential when evaluating natural medicines [21,22]. There is an urgent need to develop innovative and more rigorous quality evaluation standards for natural treatments. In this study, Herba Hyssopi was selected as the primary research subject. Samples of HCB collected from various geographical origins, its cultivated variety (*Hyssopus officinalis* L., HOL), and the common adulterant NBB were analyzed. UPLC-LTQ-Orbitrap-MS was employed to identify and characterize the non-volatile chemical constituents of HCB. In addition, to clarify the bioactive substances and mechanisms behind the antitussive and anti-asthmatic actions of HCB, molecular docking, network pharmacology, and cellular tests were combined. The ultimate objective of this work is to develop a specific HCB detection technique, enhance its quality assessment system, and offer a strong scientific basis for standardizing the quality control of formulations containing HCB as a primary component.

## 2. Results

### 2.1. Determination of the Chemical Composition of Herba Hyssopi

**Figure 1:**
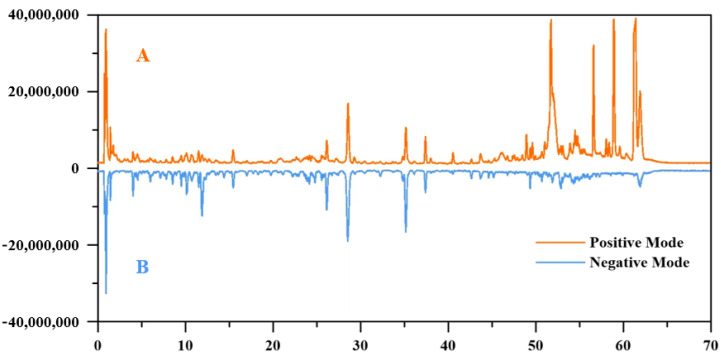
Total ion chromatograms of Herba Hyssopi in both positive (**A**) and negative (**B**) ion modes.

The chemical composition database of HCB was developed based on existing literature. A multi-parameter comparison comprising retention duration, exact molecular mass, and distinctive mass spectrum fragmentation pattern was used to identify the compound. Overall, 41 compounds were identified, comprising 14 flavonoids, 15 organic acids, 6 phenylpropanoids, 3 terpenoids, and 3 other constituents. Among these, 19 compounds were confirmed by comparison with authentic reference standards, including citric acid, danshensu, neochlorogenic acid, chlorogenic acid, cryptochlorogenic acid, ferulic acid, caffeic acid, salvianolic acid A, rutin, cynaroside, diosmin, rosmarinic acid, salvianolic acid B, luteolin, linarin, quercetin, calycosin, oleanolic acid, and ursolic acid, The mass spectrometric information of the identified chemical components obtained through UPLC-LTQ-Orbitrap-MS/MS is presented in Table 1, and the total ion chromatogram (TIC) under (±) ESI-MS mode is shown in Figure 1. The substitute, HOL, contains essentially the same types of components as HCB, but there are specific differences in the concentration levels of these components. The adulterant NBB exhibits more significant differences in its composition compared to HCB. While the variety of organic acids present shows little discrepancy, there are considerable differences in the types of flavonoids and triterpenoids.

### 2.2. Network Pharmacology

#### 2.2.1. The Collection of the Targets for the Compound and the Disease

**Figure 2:**
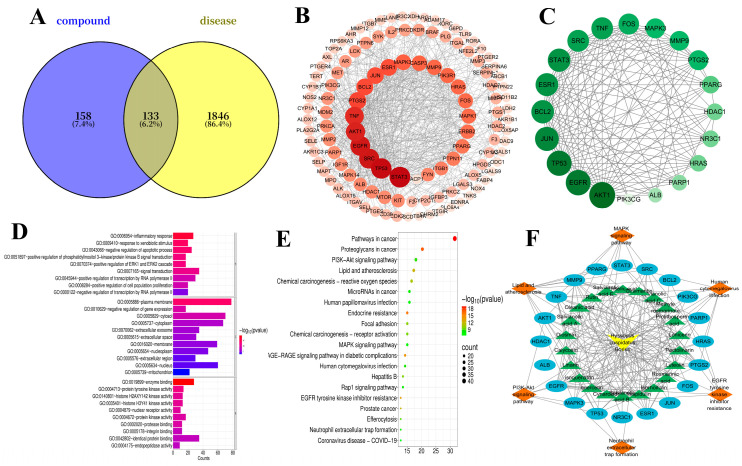
(**A**) 133 intersection targets of Herba Hyssopi treatment for cough and asthma. (**B**) These 133 common targets constructed a PPI network. (**C**) The potential therapeutic genes of Herba Hyssopi for the treatment of cough and asthma. (**D**) GO Enrichment bar graph. (**E**) KEGG bubble diagram. (**F**) “drug-component-target-pathway” network, the yellow V-shape represents drug, the green triangles represent compounds, the blue ellipses represent targets, the orange diamonds represent pathways, and the edges represent interactions between compounds and targets or between targets and pathways.

A total of 28 potential active compounds were screened from the 41 identified components of Herba Hyssopi, and subsequently, 291 targets associated with these components were collected after removing duplicates. After screening, aggregating, and removing duplicates, 1979 disease targets were obtained by GeneCards, NCBI, and DisGeNET databases. Using the Venny online platform to draw a Venny diagram, through intersection analysis [23,24], 133 common targets for Herba Hyssopi in the treatment of “cough” and “asthma” (potential therapeutic targets) were identified, as shown in Figure 2A.

#### 2.2.2. The “Protein–Protein Interaction” Networks

An interaction score of more than 0.7 was selected for constructing the PPI network after the 133 common targets were uploaded to the STRING database [25]. As shown in Figure 2B, this PPI network is visualized in Cytoscape 3.10.1. The degree values of the shapes correspond to their size and color depth. Subsequently, potential therapeutic genes were screened using CytoNCA, resulting in a network diagram composed of 20 genes that are involved in Herba Hyssopi for the treatment of cough and asthma, including AKT1, EGFR, TP53, STAT3, SRC, TNF, PTGS2, BCL2, ESR1, and JUN. Their interrelationships are illustrated in Figure 2C.

#### 2.2.3. Gene Ontology (GO) and KEGG Analysis

For GO enrichment analysis, 133 key targets were submitted to the DAVID database, and the top 10 significantly enriched GO terms were visualized as a bar chart (Figure 2D). The results indicated that the potential targets of Herba Hyssopi participate in various biological processes, including the inflammatory response, response to xenobiotic stimuli, negative regulation of apoptosis, and activation of the PI3K/Akt and ERK1/ERK2 signaling pathways.

Then, KEGG pathway enrichment analysis was performed on these key targets, and the top 15 pathways were illustrated in a bubble plot (Figure 2E). The findings suggest that the therapeutic effects of Herba Hyssopi in treating cough and asthma may involve multiple pathways, including lipid and atherosclerosis signaling, human cytomegalovirus infection, EGFR tyrosine kinase inhibitor resistance, and the PI3K–Akt and MAPK signaling pathways, among others.

#### 2.2.4. Construction of “Drug-Component-Target-Pathway” Network

A drug-component-target-pathway network was constructed to investigate the relationship between the disease and the active ingredients of Herba Hyssopi (Figure 2F). A visual key is used in the network: the drug is denoted by a yellow V-shape, compounds by green triangles, targets by blue ellipses, and pathways by orange diamonds. The edges, respectively, represent the compound-target and target-pathway interactions. The network reveals complex compound-target interactions, implying that the therapeutic effect of Herba Hyssopi arises from its components collectively modulating a network of targets, thus producing robust synergistic activity. The therapeutic efficacy of Herba Hyssopi is attained through a variety of pharmacological effects on multiple targets and pathways. Based on degree centrality analysis, targets such as EGFR (degree = 17), MMP9 (degree = 12), TNF (degree = 11), and PTGS2 (degree = 10) show high connectivity within the network, suggesting they may represent the primary targets through which Herba Hyssopi exerts its therapeutic effects on relevant diseases.

### 2.3. Molecular Docking Validation

The network pharmacology results showed that the 7 proteins of EGFR, MMP9, TNF, PTGS2, MAPK3, ESR1, and TP53 exhibited significant importance in two key aspects: first, they all displayed node degree values greater than 10 in the drug-component-target-pathway network; second, they ranked among the top 20 therapeutic targets in the PPI network. Furthermore, literature studies have confirmed that these targets are closely associated with related diseases [26,27]. So they were selected for molecular docking analysis. The compounds rosmarinic acid, quercetin, diosmin, linarin, lonicerin, and ursolic acid showed strong correlations with these targets and were therefore considered as major active constituents potentially contributing to the therapeutic effects of Herba Hyssopi in treating cough and asthma.

**Figure 3:**
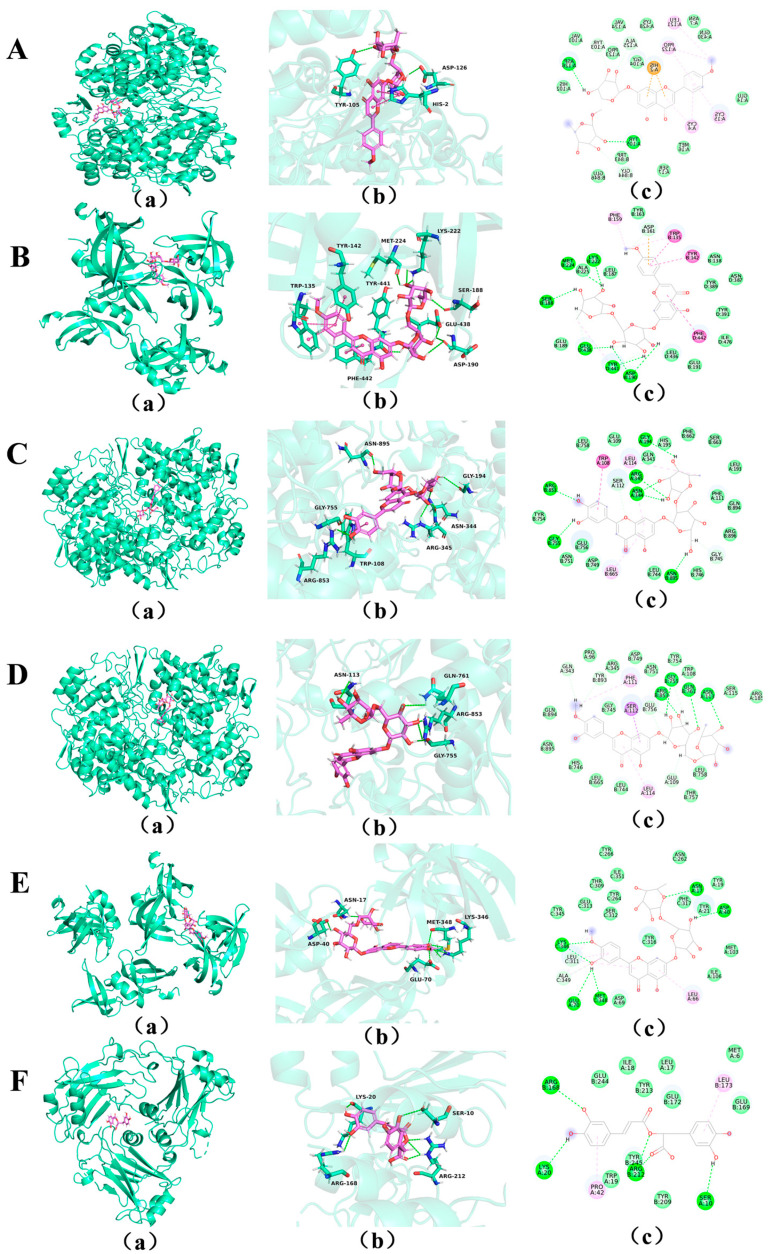
Molecular docking results of effective compounds of Herba Hyssopi with potential targets. (**A**) PTGS2 and linarin. (**B**) TP53 and linarin. (**C**) PTGS2 and lonicerin. (**D**) PTGS2 and diosmin. (**E**) TP53 and diosmin. (**F**) TNF and rosmarinic acid. (**a**) 3D molecular docking structure; (**b**) Detailed 3D molecular docking structure; (**c**) 2D molecular docking structure.

Among them, quercetin, diosmin, linarin, and lonicerin were also identified as key differential markers distinguishing HCB from its common adulterants. Molecular docking was conducted to evaluate the binding affinities between these compounds and the selected targets. Strong binding forces between protein targets and pharmacological ligands were thought to be indicated by interaction energies less than −5 kcal/mol [28]. Hydrogen bonds play an essential role in molecular docking and their quantity and characteristics directly impact both binding energy and stability. A higher number of hydrogen bonds indicates enhanced bonding stability [29]. By integrating these two significant parameters, namely binding energy and hydrogen bonding, a more comprehensive assessment can be made regarding the binding strength of compounds to core targets. The results are presented in Table 2. The docking results revealed binding energies ranging from −10.5 to −7.5 kcal/mol, indicating strong interactions. Notably, linarin, lonicerin, diosmin, and rosmarinic acid have shown strong binding affinities with TP53. Linarin had the richer hydrogen bonds formed with various proteins, and the structural conformation was more stable. Linarin may exert its therapeutic effect on cough and asthma by acting on amino acid residues such as LYS-222, GLU-438, and APS-190 of the TP53 protein and forming eight hydrogen bonds with it. Among them, the three possible docking modes of six compound–target pairs with the lower binding energy or more hydrogen bonds were selected and shown in Figure 3. Figure 3 shows the 3D simulation plots and 2D simulation plots of molecular docking, respectively, clearly showing the binding mode and 3D structure between various active components and related targets. The results showed that the four effective components (linarin, lonicerin, diosmin, and rosmarinic acid) had a strong binding ability to the three targets (TNF, PTGS2, and TP53). Considering both molecular docking outcomes and compound abundance, linarin, diosmin, and rosmarinic acid were identified as the principal active constituents. Moreover, linarin and diosmin also serve as distinguishing markers, differentiating HCB from its adulterants. Therefore, these three compounds were selected for subsequent experimental validation. Also, the results of molecular docking further cross-validated the relevant results of network pharmacology.

### 2.4. Verification Study of Diosmin, Linarin, and Rosmarinic Acid In Vitro

#### 2.4.1. Effects of Diosmin, Linarin, and Rosmarinic Acid on RAW264.7 Cell Viability

**Figure 4:**
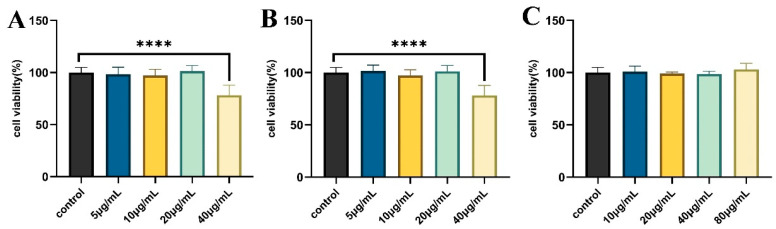
Effects of diosmin (**A**), linarin (**B**) and rosmarinic acid (**C**) on cell viability. Data are displayed as mean ± SD (*n* = 6). **** *p* < 0.0001 vs. the control group.

To assess the effects of diosmin, linarin, and rosmarinic acid on the viability of normal RAW264.7 cells, the cells were subjected to treatment with various concentrations of diosmin (5, 10, 20, and 40 μg/mL), linarin (5, 10, 20, and 40 μg/mL), and rosmarinic acid (10, 20, 40, and 80 μg/mL). Cell viability was evaluated using the CCK-8 assay. As shown in Figure 4C, there were no significant differences in cell viability between the rosmarinic acid treatment groups (10–80 μg/mL) and the control group, indicating that rosmarinic acid exerted no cytotoxic effects within this concentration range.

In comparison, treatment with 40 μg/mL diosmin (Figure 4A) or 40 μg/mL linarin (Figure 4B) significantly reduced the proliferation of RAW264.7 cells (*p* < 0.0001). Based on these results, diosmin (20 μg/mL), linarin (20 μg/mL), and rosmarinic acid (80 μg/mL) were identified as non-cytotoxic and were therefore selected for subsequent experiments.

#### 2.4.2. Impact of Different Concentrations of Diosmin, Linarin, and Rosmarinic Acid on NO Production and Pro-Inflammatory Cytokines

**Figure 5:**
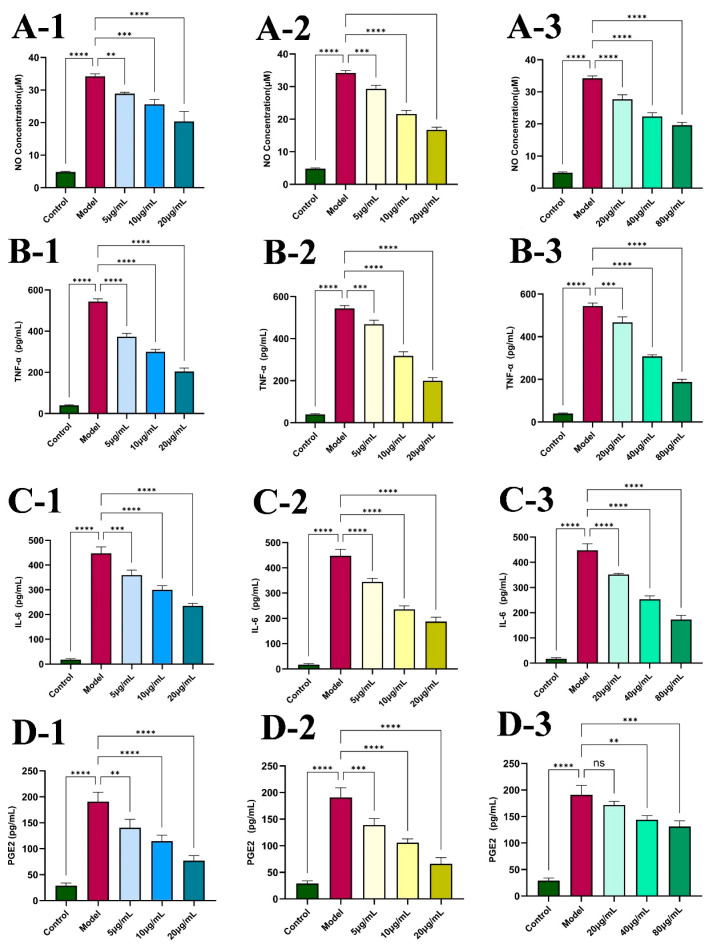
Effects of different doses of diosmin (1), linarin (2) and rosmarinic acid (3) on NO (**A**) and pro-inflammatory cytokines TNF-α (**B**), IL-6 (**C**) and PGE2 (**D**). Data are displayed as mean ± SD (n = 3). ** *p* < 0.01, *** *p* < 0.001 and **** *p* < 0.0001 vs. control or model group. (**A-1**) Effects of different doses of diosmin on NO. (**A-2**) Effects of different doses of linarin on NO. (**A-3**) Effects of different doses of rosmarinic acid on NO. (**B-1**) Effects of different doses of diosmin on TNF-α. (**B-2**) Effects of different doses of linarin on TNF-α. (**B-3**) Effects of different doses of rosmarinic acid on TNF-α. (**C-1**) Effects of different doses of diosmin on IL-6. (**C-2**) Effects of different doses of linarin on IL-6. (**C-3**) Effects of different doses of rosmarinic acid on IL-6. (**D-1**) Effects of different doses of diosmin on PGE2. (**D-2**) Effects of different doses of linarin on PGE2. (**D-3**) Effects of different doses of rosmarinic acid on PGE2.

To establish an in vitro inflammatory model, RAW264.7 cells were stimulated with 1 μg/mL LPS, and the levels of NO and inflammatory cytokines in the cell culture supernatant were subsequently measured. The model group demonstrated an evident increase in NO production, confirming successful induction of an inflammatory response (Figure 5A). Treatment with various concentrations of diosmin (Figure 5A-1), linarin (Figure 5A-2), and rosmarinic acid (Figure 5A-3) significantly suppressed LPS-induced NO release compared with the model group (*p* < 0.01).

To further evaluate the anti-inflammatory potential of diosmin, linarin, and rosmarinic acid, the levels of key pro-inflammatory cytokines, TNF-α, IL-6, and PGE_2_, were determined by ELISA. As shown in Figure 5B–D, LPS stimulation significantly increased cytokine production compared with the control group (*p* < 0.0001). Both diosmin (Figure 5B-1,C-1,D-1) and linarin (Figure 5B-2,C-2,D-2) inhibited TNF-α, IL-6, and PGE_2_ secretion in a dose-dependent manner, with statistically significant reductions (*p* < 0.01). Although rosmarinic acid at 20 μg/mL did not significantly affect PGE_2_ levels (Figure 5D-3), higher concentrations (40 and 80 μg/mL) produced significant inhibition (*p* < 0.01). Moreover, rosmarinic acid at all tested concentrations (20, 40, and 80 μg/mL) significantly reduced the secretion of TNF-α (Figure 5B-3) and IL-6 (Figure 5C-3), demonstrating strong anti-inflammatory activity (*p* < 0.001).

### 2.5. Quantification of Three Components in Herba Hyssopi

#### 2.5.1. Method Validation and Content Determination

Standard calibration curves were constructed by plotting the concentration of each compound against its corresponding peak area. As summarized in Table 3, diosmin, linarin, and rosmarinic acid showed excellent linearity within their respective detection ranges. The calculated LOD and LOQ demonstrated the high sensitivity of the developed chromatographic method. Furthermore, the method’s reliability was confirmed by satisfactory results for precision, stability, repeatability, and recovery, as presented in Table 4.

**Figure 6:**
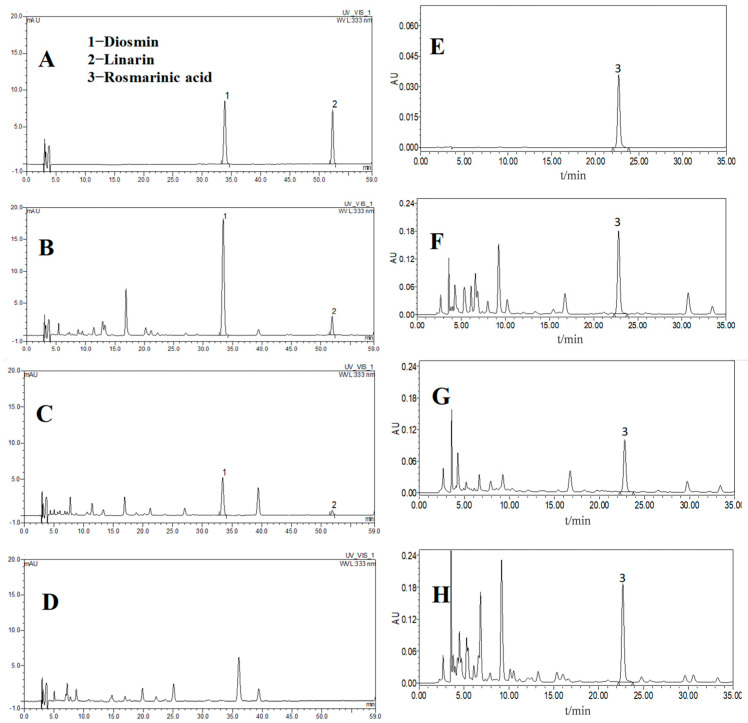
Typical HPLC chromatograms in gradient elution: mixed standard solution of diosmin and linarin (**A**), Methanolic extract of HCB (**B**) Methanolic extract of HOL (**C**), Methanolic extract of NBB (**D**); typical HPLC chromatograms in isocratic elution: standard solution of Rosmarinic acid (**E**), 70% Methanolic extract of HCB (**F**) 70% Methanolic extract of HOL (**G**), 70% Methanolic extract of NBB (**H**); peaks 1–3 were represented for diosmin, linarin, and rosmarinic acid.

Using the validated method, the contents of diosmin, linarin, and rosmarinic acid were determined in twenty Herba Hyssopi samples from different batches, with the results summarized in Table 5. Representative chromatograms of the analyzed compounds are shown in Figure 6.

#### 2.5.2. Multivariate Chemometric Analysis

**Figure 7:**
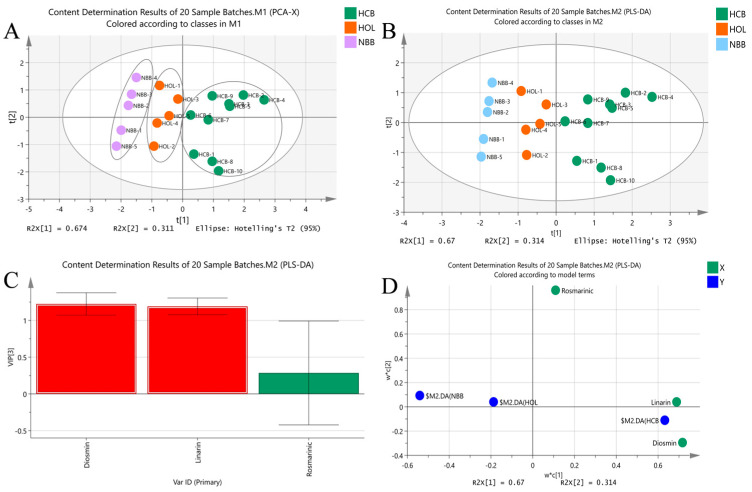
Multivariate analysis of 20 batches of Herba Hyssopi: (**A**) PCA score scatter plot; (**B**) PLS-DA score scatter plot; (**C**) VIP plot derived from PLS-DA; (**D**) Loading scatter plot generated from PLS-DA.

Unsupervised pattern recognition analysis, using PCA, a chemometric technique that reduces a matrix of data to its lowest dimension of the most significant factors, was used to clearly distinguish the quality differences in Sample from different origins. The concentration data of three key compounds from twenty batches of samples representing various botanical sources were imported into SIMCA 14.1 software to construct a two-dimensional PCA model. As illustrated in Figure 7A, the samples were clearly grouped into three distinct clusters, indicating significant compositional differences among the sample origins.

To enhance classification accuracy and minimize intra-group variability, partial least squares discriminant analysis (PLS-DA), a supervised modeling approach, was subsequently applied. As shown in Figure 7B, PLS-DA classified the twenty batches into three well-separated groups, consistent with the PCA clustering results. Cross-validation confirmed the robustness and reliability of both models, demonstrating that the identified chemical markers effectively discriminated authentic Herba Hyssopi from adulterated samples.

The VIP plot (Figure 7C) revealed that diosmin and linarin made the greatest contributions to group separation. Similarly, the loading scatter plot (Figure 7D) showed that these two compounds were located at the outer margins, further confirming their significant influence on clustering patterns. The PCA and PLS-DA analyses identified diosmin and linarin as the primary chemical components responsible for the observed quality differences among Herba Hyssopi samples.

#### 2.5.3. Hierarchical Cluster Analysis (HCA) with Heatmap

**Figure 8:**
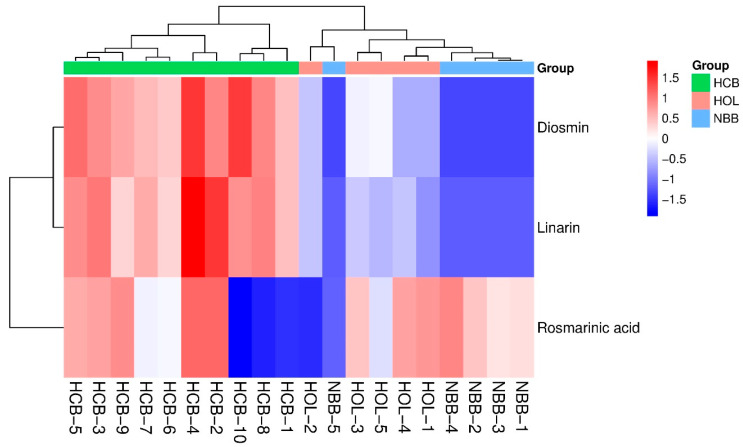
HCA of 20 batch samples with a heatmap.

The quantitative data of the three compounds from twenty sample batches were imported into MetaboAnalyst 5.0 to construct a heatmap (Figure 8). The HCA combined with heatmap visualization was employed to display the relative abundance of these constituents across samples from different botanical origins. The cluster dendrogram was generated using Euclidean distance metrics and the group-average linkage method.

As shown in the heatmap, the twenty samples were initially divided into two major clusters, with the primary intergroup differences attributed to variations in diosmin and linarin content. The highest levels of diosmin and linarin were detected in *Hyssopus cuspidatus* Boiss samples, followed by *Hyssopus officinalis* L., whereas these compounds were nearly absent in *Nepeta bracteata* Benth. Further subdivision of each cluster, based on rosmarinic acid content, produced two subgroups; however, no significant differences in rosmarinic acid levels were observed among samples from the different botanical sources.

## 3. Discussion

A growing body of evidence suggests that natural chemicals derived from ethnomedicinal herbs are becoming an essential source for the discovery of therapeutic agents in the treatment of diseases [30,31]. Historical texts indicate that Herba Hyssopi has been used in various countries and regions for the treatment of cough, asthma, edema, and other diseases, and its use continues to this day in the Xinjiang Uyghur Autonomous Region [32,33]. However, the active chemical constituents and their underlying mechanisms of action remain insufficiently understood, hindering the advancement of medicinal applications. Considering the complex multi-component and multi-target characteristics of natural medicines [34,35], it is essential to develop an effective strategy for identifying quality marker compounds that accurately represent therapeutic efficacy, therefore improving and standardizing quality evaluation systems. Here, Herba Hyssopi was selected as the research subject to identify bioactive constituents and quality markers associated with its pharmacological effects through an integrated multi-method approach. Furthermore, a quantitative analytical method was established to differentiate samples originating from different botanical sources.

In this study, a total of 41 chemical constituents were identified using UPLC-LTQ-Orbitrap-MS, comprising 14 flavonoids, 15 organic acids, 6 phenylpropanoids, 3 terpenoids, and 3 other minor compounds. The major constituents detected at relatively higher concentrations included phenolic acids such as caffeic acid, chlorogenic acid, salvianolic acid E, and rosmarinic acid; flavonoids such as linarin and diosmin; as well as triterpenoids including oleanolic acid and ursolic acid.

Modern pharmacological investigations have demonstrated that phenolic acid molecules, such as chlorogenic acid, caffeic acid, and rosmarinic acid, show numerous biological actions, including anti-inflammatory, antioxidant, anticancer, and immunomodulatory properties [36,37]. The flavonoid component diosmin has demonstrated significant therapeutic efficacy in treating hemorrhoids, lumbar spinal stenosis, and arthritis [38,39]. Linarin, a natural chemical constituent commonly found in medicinal plants such as Chrysanthemum indicum and *Buddleja officinalis*, demonstrates notable anti-inflammatory and antioxidant properties. It can decrease growth and induce apoptosis in various tumor cells [40]. Furthermore, studies have reported that linarin may alleviate airway inflammation in asthmatic mice through CAMKII/DRP1-mediated mitochondrial damage and mitophagy, demonstrating therapeutic potential for asthma [41]. These findings collectively suggest that these compounds are putative key constituents responsible for the pharmacological effects of Herba Hyssopi, thus laying the groundwork for this study.

In the field of natural products research, network pharmacology provides a powerful framework for discovering drug targets and screening promising active compounds [42,43]. The combination of network pharmacology with experiments has been recognized as a new method for detecting molecular mechanisms in natural medicines. The combined use of UPLC-LTQ-Orbitrap-MS and network pharmacology to screen the potential active ingredients in Herba Hyssopi not only represents efficacy but also reflects measurability, which is the central tenet of the five principles of Q-markers [44,45]. Furthermore, the effective components were subjected to origin and therapeutic attribution analysis. Our network pharmacology investigation identified 291 potential targets for the chemical constituents of Herba Hyssopi, with a notable 45.7% of these targets being associated with cough or asthma. In the protein–protein interaction network, AKT1, EGFR, TP53, STAT3, SRC, TNF, PTGS2, and BCL2 showed higher connectivity degrees than other nodes, suggesting that these proteins may serve as key targets through which Herba Hyssopi exerts its therapeutic effects against cough and asthma. Results from the KEGG enrichment and GO analyses indicated that the bioactive constituents of Herba Hyssopi likely mediate anti-asthmatic effects by modulating processes such as inflammatory response, apoptosis, signal transduction, and immune regulation across multiple signaling pathways. The most enriched pathways included lipid and atherosclerosis signaling, EGFR tyrosine kinase inhibitor resistance, PI3K–Akt, and MAPK signaling pathways. Moreover, the observed upregulation of the MAPK signaling pathway in bronchial asthma model rats, compared with normal controls, aligns with previously reported findings, further supporting these mechanistic insights [46]. The PI3K-Akt pathway, a typical signaling cascade, is crucial in controlling essential cellular functions, such as proliferation and apoptotic cell death [47], as well as regulating the inflammatory response [48]. According to a network study of drug-component-target-pathway sorted by degree value, EGFR, MMP9, TNF, PTGS2, MAPK3, ESR1, and TP53 are the primary targets through which Herba Hyssopi exerts pharmacological effects. Previous research has shown that the EGFR plays a crucial role in asthma-related airway inflammation and remodeling, and is closely linked to airway thickness and airway hyperresponsiveness (AHR) [49,50]. Aside from EGFR, TNF plays a crucial role in exacerbating airway inflammation. TNF-α binds to TNFR1/2, which in turn results in the activation of the MAPK and NF-κB pathways. This causes the prolonged release of downstream inflammatory molecules, such as IL-6 and IL-1β, resulting in increased airway inflammation and hyperresponsiveness [51,52,53]. In terms of airway remodeling, MMP-9 is one of the most strongly associated ECM-degrading enzymes with asthmatic remodeling. The imbalance between MMP-9 activation and its inhibitor TIMP-1 promotes degradation of the airway basement membrane, fibrosis, and structural reconstruction [54]. Plant-derived active components, such as green tea extract, can alleviate airway inflammation and remodeling by inhibiting the expression and activity of MMP-9, suggesting the potential of natural products in asthma treatment [55,56]. Based on their high association with the core proteins, we further identified six constituents (rosmarinic acid, quercetin, diosmin, linarin, lonicerin, and ursolic acid) as the core constituents of Herba Hyssopi for the treatment of cough and asthma.

Furthermore, previous studies have shown that the six substances listed above have pharmacological actions related to managing asthma or relieving cough. For example, investigations conducted both in vitro and in vivo have shown that rosmarinic acid has anti-inflammatory and antioxidant bioactivities [57]. Similarly, after LPS stimulation, quercetin has been shown to prevent cells from releasing inflammatory proteins, including TNF-α and IL-6 [58]. Diosmin can protect against LPS-induced acute lung injury in mice [59]. Linarin plays a significant part in protecting cells from oxidative stress-induced toxicity [60]. Lonicerin alleviates asthma by exerting anti-inflammatory and immunomodulatory effects through inhibition of the SRC/EGFR pathway [61]. And ursolic acid demonstrates pharmacological activities, including antimicrobial, anti-inflammatory, and anticancer effects [62].

Molecular docking analysis was conducted to evaluate the binding interactions between the six major active constituents of Herba Hyssopi and the seven previously identified target proteins. It is well established that lower binding energy values correspond to stronger ligand–receptor interactions and greater complex stability. Typically, a binding energy below 5 kcal/mol is considered indicative of meaningful binding activity. The results showed that all compound–target interactions demonstrated binding energies below this threshold, suggesting favorable and stable affinities between the six compounds and the seven targets. Both hydrogen bonding and hydrophobic interactions were found to play key roles in stabilizing the protein–ligand complexes. In biological systems, hydrogen bonds are particularly significant, not only for maintaining the structural integrity of protein–ligand interactions but also for facilitating a wide range of physiological processes [63]. The molecular docking results revealed that the protein targets PTGS2, TP53, TNF, and MAPK3 demonstrated the most favorable binding affinities with the active compounds. Among these, linarin, lonicerin, diosmin, and rosmarinic acid demonstrated the strongest binding interactions with the four key targets. Considering both their binding affinities and relative abundance in Herba Hyssopi, linarin, diosmin, and rosmarinic acid were tentatively identified as the principal active constituents responsible for its therapeutic effects against cough and asthma. However, further experimental studies are warranted to validate these findings. The core molecular targets implicated in the pharmacological action of Herba Hyssopi include PTGS2, TP53, TNF, and MAPK3. TNF acts as a key receptor that promotes the expression and release of inflammatory mediators such as IL-6, contributing to excessive mucus secretion and airway inflammation characteristic of asthma [64].

We examined the effects of linarin, diosmin, and rosmarinic acid on the inflammatory factors TNF-α, IL-6, and PGE2 in an LPS-induced RAW 264.7 cell culture to corroborate the findings of network pharmacology and molecular docking. The findings demonstrated that rosmarinic acid, linarin, and diosmin may considerably lower the levels of inflammatory factors. In other words, Herba Hyssopi’s strong anti-inflammatory properties, which are mediated by important anti-inflammatory substances such as diosmin, linarin, and rosmarinic acid, are likely responsible for its antitussive and anti-asthmatic benefits.

Building upon the identification of key bioactive constituents, this study successfully established a robust HPLC-based quality control method for Hyssopus officinalis. The developed technique enabled the simultaneous quantification of three characteristic compounds, demonstrating excellent linearity between peak area and concentration across the tested ranges. The method showed satisfactory recovery, stability, precision, and repeatability, all of which met the predefined validation criteria. The established protocol is simple, accurate, cost-effective, and efficient. This approach represents a methodological advancement in the quality evaluation of natural medicines, shifting from single-component to multi-component analysis and from marker-based to efficacy-oriented quality assessment. Using this validated method, the concentrations of the three target compounds were determined in 10 batches of HCB, 5 batches of HOL, and 5 batches of NBB. Multivariate chemometric analysis revealed that the quantification of diosmin and linarin effectively differentiated these three medicinal sources.

The origin identification approach developed in this study provides a reliable analytical alternative to traditional morphological and microscopic identification methods. Its accuracy and practicality make it broadly applicable for standardizing the authentication of natural medicine, while addressing the current challenges of limited expertise and declining technical knowledge in traditional identification practices.

## 4. Materials and Methods

### 4.1. Reagents and Materials

Samples were provided by the Xinjiang Uygur Autonomous Region Institute for Drug Inspection and Research and authenticated by Amatjan Ayupbek, Chief Pharmacist of the institute. The collection included 10 batches of *Hyssopus cuspidatus* Boiss. (HCB), 5 batches of *Hyssopus officinalis* L. (HOL), and 5 batches of *Nepeta bracteata* Benth. (NBB). The National Institutes for Food and Drug Control (Beijing, China) provided reference standards for diosmin (purity 92.2%, code 101348-202203), linarin (purity 98.5%, code 111528-201911), and rosmarinic acid (purity 98.1%, code 111871-202007).

The American Type Culture Collection (ATCC, Manassas, VA, USA) provided the RAW 264.7 murine macrophage cell line. Lipopolysaccharide (LPS) derived from *Escherichia coli* was supplied by Sigma-Aldrich (St. Louis, MO, USA). Dulbecco’s Modified Eagle Medium (DMEM), fetal bovine serum (FBS), and penicillin–streptomycin solution were obtained from Gibco (Grand Island, NY, USA). The nitric oxide (NO) assay kit and ELISA kits for mouse TNF-α, IL-6, and PGE_2_ were procured from Beyotime Biotechnology (Shanghai, China).

Methanol and acetonitrile were chromatographically pure (Merck, Darmstadt, Germany); water was purified water (Watsons distilled drinking water, Guangzhou, China); formic acid was chromatographically pure (Macklin, Shanghai, China); all other reagents were of analytical grade. Helium gas with a purity of 99.999% (*v*/*v*), purchased from Shijiazhuang Xisanjiang Practical Gas Co., Ltd. (Shijiazhuang, China), served as the collision gas in the DART system. Nitrogen gas with a purity of 99.999% (*v*/*v*), generated by a nitrogen generator from Peak Gas Generators (Billerica, MA, USA), served as the carrier gas.

### 4.2. The Detection of Chemical Compositions in Herba Hyssopi

#### 4.2.1. Preparation of the Herba Hyssopi Extraction Solution

Approximately 0.5 g of the sieved (No. 3 sieve) sample powder was accurately weighed and transferred into a stopper cone-shaped flask. Then, 50 mL of 80% methanol had been precisely added, and the flask had been tightly sealed before the weight had been determined. The mixture was sonicated in an ultrasonic water bath (40 kHz, 500 W) for 30 min, then cooled to room temperature and reweighed. Any solvent loss was compensated for with 80% methanol. A 0.22 μm microporous membrane was used to filter the mixture, and the filtrate was employed as the test solution for qualitative analysis.

#### 4.2.2. UPLC-LTQ-Orbitrap-MS Condition

An Ultimate 3000 UPLC system and an LTQ-Orbitrap mass spectrometer (Thermo Fisher Scientific, Waltham, MA, USA) were used for chromatographic separation. A Waters ACQUITY UPLC BEH C18 column (2.1 × 100 mm, 1.7 μm) maintained at 30 °C was employed for analysis. The mobile phase consisted of acetonitrile (A) and 0.1% formic acid in water (B), at a flow rate of 0.4 mL/min and an injection volume of 3 μL. The gradient elution was programmed as follows: 0–20 min, 5–55% A; 20–35 min, 55–65% A; 35–60 min, 65–90% A; 60–62 min, 90% A; 62–63 min, 90–5% A; and 63–66 min, 5% A.

The LTQ-Orbitrap system was used for high-resolution data-dependent acquisition (DDA) mass spectrometric detection. The following parameters were used to run the ionization source in both positive and negative electrospray ionization (ESI±) modes: sheath gas flow rate of 50 arb; auxiliary gas flow rate of 10 arb; ion source temperature of 350 °C; and capillary voltage of 3.5 kV/3.0 kV (positive/negative). Complete scan: MS data were collected in both positive and negative polarities over an m/z range of 150–1500. Data acquisition and processing were performed using Xcalibur 2.1 software (Thermo Fisher Scientific, Waltham, MA, USA).

### 4.3. Network Pharmacology Analysis

#### 4.3.1. Compound and Disease Targets Prediction

The chemical constituents identified by UPLC-LTQ-Orbitrap-MS were further analyzed using a network pharmacology approach. Considering the indirect relationship between the bioactive components of traditional Chinese medicine (TCM) and their bioavailability [65,66], compounds with a drug-likeness (DL) value ≥ 0.18 were selected as the potential active ingredients of Herba Hyssopi. The preferred compounds were entered into the PubChem database (https://pubchem.ncbi.nlm.nih.gov/) (accessed on 14 March 2025). [67] to obtain their standardized SMILES structures. Potential target proteins of these active compounds were predicted targets of active compounds were obtained from databases such as Swiss Target Predictionusing the SwissTargetPrediction database (http://swisstargetprediction.ch/) (accessed on 14 March 2025) [68], and only targets with a probability greater than zero were retained for further analysis.

To identify disease-related genes, the keywords “cough” and “asthma” were searched in disease-associated databases, including GeneCards (https://www.genecards.org/ (accessed on 17 March 2025); results filtered using the median method to retain those with a relevance score > 1.2805), DisGeNET (https://www.disgenet.org/) (accessed on 17 March 2025), and OMIM (Online Mendelian Inheritance in Man, https://omim.org/) (accessed on 17 March 2025). The retrieved data from these sources were combined, and overlapping targets between the compound-related and disease-related genes were identified using the Venny tool. These common targets were considered potential therapeutic targets for Herba Hyssopi in the management of asthma and cough.

#### 4.3.2. Network Construction

To identify potential targets for asthma and cough therapy, we constructed a PPI network. The PPI network was built by establishing parameters and constraints employing the STRING database (https://cn.string-db.org/) (accessed on 18 March 2025) [69,70,71]. Interactions were screened using a higher confidence threshold of 0.7 as the minimum necessary interaction score, and the species was restricted to *Homo sapiens*. The PPI network was then further analyzed and modified using the CytoNCA plugin for Cytoscape 3.10.1. We identified the top 20 nodes based on degree using the CytoNCA plugin.

#### 4.3.3. GO and KEGG Enrichment Analysis

The GO and KEGG pathway enrichment analysis was performed using the DAVID database (https://davidbioinformatics.nih.gov/) (accessed on 18 March 2025) based on the PPI results [72], with an emphasis on results with a *p*-value < 0.01. The filtered results were shown using the online bioinformatics platform (http://www.bioinformatics.com.cn/) (accessed on 18 March 2025). The top 20 relevant KEGG pathways and the top 30 GO enrichments (10 Molecular Functions, 10 Cellular Components, and 10 Biological Processes) were used to create bubble plots and enrichment bar charts.

#### 4.3.4. Establishment of “Drug-Component-Target-Pathway” Network

To clarify the complex interactions among drug components, targets, and signaling pathways, and to identify key bioactive compounds and potential core targets through network topology analysis, Cytoscape 3.10.1 software was employed to construct a comprehensive drug–component–target–pathway network. The network was generated by importing the relevant network and attribute tables, where nodes represented drugs, active compounds, targets, and pathways, and edges indicated the interactions among these elements. Topological characteristics, such as degree, betweenness centrality, and closeness centrality, were computed using Cytoscape’s built-in “Network Analyzer” tool. These results were then utilized to determine the network’s critical targets.

### 4.4. Molecular Docking

To further validate the interactions between active compounds and their target proteins, molecular docking analysis was performed. The 3D structures (SDF format) of the active compounds were retrieved from the PubChem database (https://pubchem.ncbi.nlm.nih.gov/). The crystal structures of EGFR (PDB:9BY6), MMP9 (PDB:8K5V), TNF (PDB:7YPC), PTGS2 (PDB:3OLT), MAPK3 (PDB:2ZOQ), ESR1 (PDB:6PSJ), and TP53 (PDB:8SWJ) were obtained from the RCSB Protein Data Bank (https://www.rcsb.org/) (accessed on 20 March 2025). Using PyMOL 3.0, the co-crystallized ligand, water molecules, and any heteroatoms not relevant to the binding site were removed. The protein structure was then prepared using AutoDockTools 4 by adding polar hydrogen atoms and assigning Gasteiger charges. Docking simulations were carried out using AutoDock Vina 1.2.5 [73,74,75], sequentially docking the ligands to the protein and calculating the binding energies. The grid box was centered on the binding pocket as defined by the native ligand in the PDB structure, with the following dimensions: EGFR (center_ x = −4.75, center_ y = −57.08, center_ z = 22.95; size_ x = 71.04, size_ y = 52.59, size_ z = 62.53), MMP9 (center_ x = 21.29, center_ y = 0.06, center_ z = 9.04; size_ x = 62.32, size_ y = 50.38, size_ z = 87.58), TNF (center_ x = −7.77, center_ y = −7.7, center_ z = −48.42; size_ x = 76.96, size_ y = 77.12, size_ z = 76.54), PTGS2 (center_ x = 25.24, center_ y = 27.30, center_ z = 47.31; size_ x = 80.88, size_ y = 79.51, size_ z = 104.57), MAPK3 (center_ x = 16.32, center_ y = −9.63, center_ z = 23.47; size_ x = 89.83, size_ y = 73.12, size_ z = 106.71), ESR1 (center_ x = 14.16, center_ y = −25.29, center_ z = 19.57; size_ x = 67.62, size_ y = 63.53, size_ z = 57.96), TP53 (center_ x = 22.61, center_ y = −18.13, center_ z = 20.87; size_ x = 82.26, size_ y = 75.48, size_ z = 71.5). A grid spacing of 0.375 Å was used. The docking calculations utilized the default scoring function implemented in AutoDock Vina. To ensure the robustness of the docking poses, the exhaustiveness parameter was set to 32. For each ligand, 10 independent docking runs were performed. After docking was completed, the docking conformations were ranked based on their scores, and the optimal structure was selected for analysis. The ligand-receptor binding affinity, quantified by interaction energy and binding modes, was calculated. The docking poses with the most favorable binding affinity were visualized and analyzed using PyMOL 3.0 software.

### 4.5. In Vitro Verification of Predicted Targets and Mechanisms

#### 4.5.1. Cell Culture

RAW264.7 cells were cultured at 37 °C in a humidified 5% CO_2_ environment using DMEM supplemented with 10% fetal bovine serum (FBS) and 1% penicillin-streptomycin. When the cells reached approximately 80% confluence, subculturing was performed.

#### 4.5.2. Detection of Cell Toxicity by the CCK-8 Method

Cells in the logarithmic growth phase were seeded into 96-well plates at a density of 1 × 10^2^ cells per well to assess the inhibitory effects of diosmin, linarin, and rosmarinic acid on RAW264.7 cell proliferation. The cells were then treated with varying concentrations of diosmin (5, 10, 20, and 40 μg/mL), linarin (5, 10, 20, and 40 μg/mL), and rosmarinic acid (10, 20, 40, and 80 μg/mL). Untreated cells served as the blank control group. Following a 24 h incubation period, each well received CCK-8 reagent (10 μL) and was then subjected to incubation for an additional hour under standard CO_2_ conditions. Cell viability was then assessed by measuring the absorbance at 450 nm using a microplate reader.

#### 4.5.3. Detection of NO Levels

To further elucidate the mechanisms of action of diosmin, linarin, and rosmarinic acid, the levels of nitric oxide (NO) in the culture supernatant were quantified. RAW264.7 cells were seeded into 24-well plates at a density of 1 × 10^6^ cells per well and allowed to adhere overnight. An inflammatory model was then established by stimulating the cells with 1 μg/mL LPS. The experimental design included the following groups: control (untreated), model (LPS only), low-dose (LPS + 5 μg/mL diosmin, 5 μg/mL linarin, or 20 μg/mL rosmarinic acid), medium-dose (LPS + 10 μg/mL diosmin, 10 μg/mL linarin, or 40 μg/mL rosmarinic acid), and high-dose (LPS + 20 μg/mL diosmin, 20 μg/mL linarin, or 80 μg/mL rosmarinic acid). After 24 h of incubation, the culture medium was carefully aspirated, and the cell-free supernatant was collected for NO quantification, following the instructions provided with the NO assay kit.

#### 4.5.4. Quantification of Inflammatory Factor Levels

Macrophages were divided into three treatment groups: control, model, and diosmin linarin or rosmarinic acid. Cell culture supernatants were collected and examined according to the specific ELISA kit instructions to determine TNF-α, IL-6, and PGE2 levels.

### 4.6. Establishment of Quality Control Method

#### 4.6.1. HPLC Conditions

Chromatographic analysis was performed using an Ultimate 3000 HPLC system (Thermo Fisher Scientific, Germering, Germany) and a Waters e2695 HPLC system (Waters, Singapore), equipped with a Waters Symmetry C18 column (250 mm × 4.6 mm, 5 μm). For the quantitative determination of diosmin and linarin, the mobile phase consisted of acetonitrile (A) and 0.1% phosphoric acid aqueous solution (B), with the following gradient program: 0–8 min, 15% A; 8–12 min, 15–18% A; 12–15 min, 18% A; 15–25 min, 18–20% A; 25–30 min, 20% A; 30–40 min, 20–24% A; 40–45 min, 24–26% A; and 45–55 min, 26% A.

For the quantification of rosmarinic acid, an isocratic elution was applied using 21% acetonitrile (A) for 0–30 min. The column temperature was set at 30 °C, the injection volume was 10 μL, and the flow rate was kept at 1.0 mL/min. Detection wavelengths were 333 nm for diosmin and linarin, and 328 nm for rosmarinic acid.

#### 4.6.2. Preparation of Standard Solutions

An appropriate amount of each reference standard was weighed, followed by dissolution in methanol to prepare a mixed standard solution containing 10.773 μg/mL of diosmin and 3.2473 μg/mL of linarin. Separately, a rosmarinic acid reference solution was prepared by accurately weighing the standard and dissolving it in 70% methanol to attain a final concentration of 116.28 μg/mL. A 0.22 μm microporous membrane was used to filter all solutions, and the filtrates were collected as the reference standard solutions for analysis.

#### 4.6.3. Preparation of Test Solutions

A 0.1 g portion of the sample powder was accurately weighed, mixed with 100 mL of methanol, and subjected to reflux extraction at 85 °C for 50 min. After cooling, the filtration of the extract was carried out while employing a microporous membrane (0.45 μm). This was followed by the collection of the filtrate used as the test solution for diosmin and linarin.

Similarly, 0.2 g of the sample powder was accurately weighed, mixed with 50 mL of 70% methanol, and refluxed at 85 °C for 40 min. The filtration of the extract was carried out while employing a microporous membrane (0.45 μm). This was followed by the collection of the filtrate used as the rosmarinic acid test solution.

#### 4.6.4. System Suitability Test

Accurately pipette the reference standard solution and the test solution, then inject them for analysis under the specified chromatographic conditions and record the resulting chromatograms.

#### 4.6.5. Method Validation

The standard solutions were serially diluted with methanol or 70% methanol to prepare a series of concentrations for constructing the calibration curve. The final concentration ranges were as follows: diosmin (1.0773, 2.1546, 5.3865, 21.546, and 107.73 μg/mL), linarin (0.32473, 0.64946, 1.62365, 6.4946, and 32.473 μg/mL), and rosmarinic acid (1.1628, 2.3256, 5.8140, 23.256, and 116.28 μg/mL). Plotting the peak area against the corresponding standard concentration generated calibration curves. The limits of detection (LOD) and quantification (LOQ) were determined at signal-to-noise (S/N) ratios of 3 and 10, respectively.

Method precision was assessed by analyzing six replicates of the working standard solution within a single day to determine intraday precision. Stability was evaluated by analyzing the same sample solution (HCB-3) at 0, 1, 2, 4, 8, 12, 16, and 24 h.

To determine the accuracy of the quantitative method, a recovery test was performed. Six Herba Hyssopi samples of known concentration were spiked with standard solutions of diosmin, linarin, and rosmarinic acid at approximately a 1:1 ratio. The recovery rate and relative standard deviation (RSD) were then calculated to assess the method’s accuracy and reliability.

#### 4.6.6. Quantitative Analysis

Using the established method, the content of diosmin, linarin, and rosmarinic acid was determined in twenty collected sample batches, comprising ten of HCB, five of HOL, and five of NBB.

### 4.7. Statistical Analysis

GraphPad Prism version 10.5.0 was employed for all statistical analyses. The mean ± standard error of the mean (SEM) is used to display the data. One-way analysis of variance (ANOVA) and Tukey’s post hoc test were used to assess differences between various groups; *p* < 0.05 was deemed statistically significant [76]. In addition, the quantitative data of the three compounds were further analyzed using principal component analysis (PCA) and partial least squares (PLS) analysis with SIMCA 14.1 software.

## 5. Conclusions

In this study, UPLC-LTQ-Orbitrap-MS combined with network pharmacology was employed to identify potential active compounds and their corresponding therapeutic targets. Key constituents for quality control were further verified through molecular docking analysis and cellular experiments. By examining these pharmacodynamically relevant components, quality differences among Herba Hyssopi samples from different botanical origins were effectively distinguished.

This integrative strategy provides an efficacy-oriented framework for establishing quality control standards for Herba Hyssopi, supporting accurate origin identification, preventing adulteration, and promoting market standardization. Moreover, it exemplifies a pioneering approach to advancing the principle of “deep investigation and practical standardization” in the development of quality standards for traditional Chinese medicines.

## Figures and Tables

**Table 1:** Components identified in Herba Hyssopi.

No	RT (min)	Compound	Molecular Formula	Ion Source Model	Measured Value	Theoretical Value	Mass Error (ppm)	MS^2^ Fragments
1	1.42	Citric Acid	C_6_H_8_O_7_	ESI−	191.0195	191.0197	−1.0	173.0096, 146.9392, 111.0093
2	3.98	Danshensu	C_9_H_10_O_5_	ESI−	197.0450	197.0455	−2.5	179.0357, 135.0458, 72.9937
3	5.85	Neochlorogenic acid	C_16_H_18_O_9_	ESI−	353.0881	353.0878	0.8	191.0567, 179.0356, 135.0456
4	8.53	p-Coumaric acid hexose	C_15_H_18_O_8_	ESI−	325.0935	325.0928	2.2	163.0405, 119.0506
5	9.52	Chlorogenic acid	C_16_H_18_O_9_	ESI−	353.0883	353.0878	1.4	191.0566, 179.0355, 135.0457
6	9.8	Cryptochlorogenic acid	C_16_H_18_O_9_	ESI−	353.0885	353.0878	2.0	191.0567, 179.0357
7	10.12	Ferulic acid	C_10_H_10_O_4_	ESI−	193.0502	193.0506	−2.1	178.0277, 149.0614, 134.0379
8	10.55	Caffeic acid	C_9_H_8_O_4_	ESI−	179.0345	179.0349	−2.2	135.0457
9	11.51	Tuberonic acid glucoside	C_18_H_28_O_9_	ESI−	387.1664	387.1660	1.0	207.1033, 163.1134, 369.1556
10	11.84	Sinapaldehyde	C_11_H_12_O_4_	ESI−	207.0668	207.0662	2.9	192.0435, 174.0330, 163.0408
11	13.42	5-O-p-Coumaroylquinic acid	C_16_H_18_O_8_	ESI−	337.0930	337.0928	0.6	191.0567, 163.0407
12	14.66	3-O-Feruloylquinic acid	C_17_H_20_O_9_	ESI+	369.1177	369.1180	−0.81	193.0859
13	15.58	Loliolide	C_11_H_16_O_3_	ESI+	197.1174	197.1172	1.01	197.1067, 169.0859, 161.0961
14	22.15	Salvianolic acid I	C_27_H_22_O_12_	ESI−	537.1044	537.1038	1.1	493.0968, 295.0537, 313.0821
15	22.27	Salvianolic acid A isomer	C_26_H_22_O_10_	ESI−	493.1148	493.1140	1.6	295.0695, 313.0806, 203.0322
16	22.98	Hydrated salvianolic acid I	C_27_H_24_O_13_	ESI−	555.1147	555.1144	0.5	537.1241, 357.0720, 313.0804
17	24.03	Rutin	C_27_H_30_O_16_	ESI−	609.1468	609.1461	1.1	301.0443, 300.0365, 271.0179, 343.0564
18	24.09	Lonicerin	C_27_H_30_O_15_	ESI−	593.1517	593.1511	1.0	285.0480
19	24.20	Vicenin 2	C_27_H_30_O_15_	ESI+	595.1655	595.1657	−0.34	449.1077, 287.0543
20	24.54	Cynaroside	C_21_H_20_O_11_	ESI−	447.0936	447.0932	0.9	285.0409
21	24.59	Isoquercitrin	C_21_H_20_O_12_	ESI−	463.0886	463.0881	1.1	301.0361, 300.0284, 343.0468
22	24.79	Salviaflaside	C_24_H_26_O_13_	ESI−	521.1308	521.1300	1.5	359.0881, 323.0867, 161.0243
23	25.98	Salvianolic acid E	C_36_H_30_O_16_	ESI−	717.1475	717.1461	2.0	519.0950, 475.1049, 339.0517
24	26.10	Diosmin	C_28_H_32_O_15_	ESI−	607.1670	607.1668	0.3	299.0559, 284.0327
25	26.65	Isorhoifolin	C_27_H_30_O_14_	ESI−	577.1565	577.1562	0.5	269.0525
26	28.54	Rosmarinic acid	C_18_H_16_O_8_	ESI−	359.0776	359.0772	1.1	161.0250, 179.0355, 197.0460
27	34.44	Pectolinarin	C_29_H_34_O_15_	ESI+	623.1971	623.1970	0.16	477.1393, 315.0856
28	34.88	Salvianolic acid B	C_36_H_30_O_16_	ESI−	717.1468	717.1461	1.0	519.1135, 321.0501
29	35.16	Rabdosiin	C_36_H_30_O_17_	ESI−	717.1467	717.1461	0.8	519.1122, 339.0609, 321.0491
30	36.09	Methyle rosmarinate	C_19_H_18_O_8_	ESI−	373.0931	373.0928	0.8	161.0250, 211.0616, 179.0355
31	37.31	Luteolin	C_15_H_10_O_6_	ESI−	285.0411	285.0404	2.5	285.0410, 241.0515, 175.0408
32	37.39	Linarin	C_28_H_32_O_14_	ESI−	591.1722	591.1719	0.5	447.1285, 283.0694
33	37.39	Quercetin	C_15_H_10_O_7_	ESI−	301.0355	301.0353	0.7	178.9993, 151.0043
34	43.81	Hispidulin	C_16_H_12_O_6_	ESI−	299.0567	299.0561	2.0	284.0333, 271.0255
35	45.08	Prolithospermic acid	C_18_H_14_O_8_	ESI−	357.0620	357.0615	1.4	313.0727, 225.0566, 121.0301, 269.0823
36	45.15	Salvianolic acid C	C_26_H_20_O_10_	ESI−	491.0989	491.0983	1.2	311.0475, 267.0601, 179.0353, 341.0780
37	46.69	Cirsimaritin	C_17_H_14_O_6_	ESI+	315.0861	315.0863	−0.63	300.0630, 282.0524
38	48.99	Calycosin	C_16_H_12_O_5_	ESI+	285.0754	285.0757	−1.05	270.0525, 242.0583
39	56.51	Dibutyl Phthalate	C_16_H_22_O_4_	ESI+	279.1590	279.1590	0.00	149.0232, 205.0864
40	61.90	Oleanic acid	C_30_H_48_O_3_	ESI+	457.3674	457.3676	−0.44	456.4439, 411.3625, 393.3528, 203.1799
41	61.91	Ursolic acid	C_30_H_48_O_3_	ESI+	457.3673	457.3676	−0.66	411.3628, 393.3533, 297.2577, 203.1797, 163.1484

**Table 2:** Results of molecule docking.

No.	Key Target	PDB ID	Parameter	Rosmarinic Acid	Quercetin	Diosmin	Linarin	Lonicerin	Ursolic Acid
1	EGFR	9BY6	Affinity/(kcal/mol)	−8.6	−8.3	−9.1	−8.7	−8.7	−7.8
			H-bonds	4	4	2	5	4	0
2	MMP9	8K5V	Affinity/(kcal/mol)	−8.7	−8.3	−8.5	−9.1	−8.4	−8.3
			H-bonds	3	3	5	4	2	0
3	TNF	7YPC	Affinity/(kcal/mol)	−9.8	−8.1	−9.9	−9.3	−9.5	−7.8
			H-bonds	6	3	4	3	4	2
4	PTGS2	3OLT	Affinity/(kcal/mol)	−8.7	−8.4	−10.1	−10.5	−10.4	−8.8
			H-bonds	2	4	5	2	7	1
5	MAPK3	2ZOQ	Affinity/(kcal/mol)	−9.0	−7.9	−9.7	−9.8	−9.4	−8.3
			H-bonds	6	4	3	5	3	0
6	ESR1	6PSJ	Affinity/(kcal/mol)	−8.5	−7.5	−8.3	−9.1	−8.9	−8.1
			H-bonds	3	2	5	4	7	2
7	TP53	8SWJ	Affinity/(kcal/mol)	−9.9	−8.1	−9.9	−10.4	−10.2	−8.5
			H-bonds	5	3	7	8	2	0

**Table 3:** Regression equations, correlation coefficients, linearity ranges, LODs and LOQs of three components.

No.	Compound	Regression Equation	Linearity Range (ug/mL)	Correlation Coefficient (r)	LOD (μg/mL)	LOQ (μg/mL)
1	Diosmin	y = 19,678x + 107.05	1.0773–107.73	0.9999	0.0119	0.0357
2	Linarin	y = 26,536x − 2094.3	0.32473–32.473	0.9998	0.0108	0.0325
3	Rosmarinic acid	y = 6961.9x + 450.68	1.1628–116.28	0.9999	0.0387	0.116

**Table 4:** Precision, stability, repeatability and recovery of three components.

No.	Compound	Precision RSD (%) (n = 6)	Stability RSD (%) (n = 6)	Reproducibility RSD (%) (n = 6)	Recovery (n = 6) Mean ± RSD (%)
1	Diosmin	0.6	1.1	1.0	98.3 ± 1.5
2	Linarin	0.7	1.4	0.9	100.2 ± 0.7
3	Rosmarinic acid	0.3	1.2	1.2	99.5 ± 1.2

**Table 5:** Quantitative results of three constituents assayed in 20 batches of Herba Hyssopi samples.

NO	Origin	Diosmin (mg/g)	Linarin (mg/g)	Rosmarinic Acid (mg/g)
HCB-1	*Hyssopus cuspidatus* Boriss.	14.87	1.96	1.49
HCB-2	*Hyssopus cuspidatus* Boriss.	18.32	3.13	3.31
HCB-3	*Hyssopus cuspidatus* Boriss.	17.86	2.59	3.01
HCB-4	*Hyssopus cuspidatus* Boriss.	22.83	3.63	3.31
HCB-5	*Hyssopus cuspidatus* Boriss.	19.73	2.41	2.97
HCB-6	*Hyssopus cuspidatus* Boriss.	14.14	1.81	2.50
HCB-7	*Hyssopus cuspidatus* Boriss.	15.19	2.14	2.47
HCB-8	*Hyssopus cuspidatus* Boriss.	18.23	2.53	1.41
HCB-9	*Hyssopus cuspidatus* Boriss.	16.49	1.80	3.12
HCB-10	*Hyssopus cuspidatus* Boriss.	22.51	2.39	1.21
HOL-1	*Hysspous officinais* L.	5.76	0.46	3.08
HOL-2	*Hysspous officinais* L.	7.25	0.89	1.47
HOL-3	*Hysspous officinais* L.	10.22	0.97	2.83
HOL-4	*Hysspous officinais* L.	5.78	0.92	3.01
HOL-5	*Hysspous officinais* L.	10.39	0.77	2.35
NBB-1	*Nepeta bracteata* Benth.	0	0	2.69
NBB-2	*Nepeta bracteata* Benth.	0	0	2.83
NBB-3	*Nepeta bracteata* Benth.	0	0	2.67
NBB-4	*Nepeta bracteata* Benth.	0	0	3.15
NBB-5	*Nepeta bracteata* Benth.	0	0	2.73

## Data Availability

Data will be made available on request.

## Abbreviations

The following abbreviations are used in this manuscript:

AKT1	Protein Kinase B1
BCL2	B-cell lymphoma 2
CCK-8	Cell Counting Kit-8
DMEM	Dulbecco’s Modified Eagle’s Medium
ECM	extracellular matrix
EGFR	epidermal growth factor receptor
ELISA	enzyme-linked immunosorbent assay
ESR1	estrogen receptor 1
ESI	electron spray ionization
FBS	fetal bovine serum
GO	gene ontology
HCB	Hyssopus cuspidatus Boiss
HOL	*Hyssopus officinalis* L.
IL-6	interleukins-6
JUN	Jun Proto-Oncogene
KEGG	Kyoto Encyclopedia of Genes and Genomes
LPS	lipopolysaccharide
MAPK3	mitogen-activated protein kinase 3
MMP9	matrix metallopeptidase 9
NBB	Nepeta bracteata Benth
NF-κB	Nuclear Factor Kappa-Light-Chain-Enhancer of Activated B Cells
NO	nitric oxide
PGE2	prostaglandin E2
PI3K-Akt	The phosphoinositide 3-kinase-Protein Kinase B
PPI	protein–protein interaction
PTGS2	prostaglandin-endoperoxide synthase 2
SRC	Tyrosine-Protein-Kinase Src
STAT3	signal transducers and activators of transcription
TIC	total ion chromatogram
TNF	tumor necrosis factor
TNF-α	tumor necrosis factor-α
TNFR 1/2	tumor necrosis factor receptor 1/2
TP53	Tumor Protein 53
UPLC-LTQ-Orbitrap-MS	ultra-high performance liquid chromatography coupled with linear trap quadrupole-Orbitrap mass spectrometry
